# Conjugative plasmid pAW63 brings new insights into the genesis of the *Bacillus anthracis *virulence plasmid pXO2 and of the *Bacillus thuringiensis *plasmid pBT9727

**DOI:** 10.1186/1471-2164-6-103

**Published:** 2005-07-26

**Authors:** Géraldine A Van der Auwera, Lars Andrup, Jacques Mahillon

**Affiliations:** 1 Laboratory of Food and Environmental Microbiology, Université catholique de Louvain, Croix du Sud 2/12, B-1348 Louvain-la-Neuve, Belgium; 2 National Institute of Occupational Health, Lersø Parkallé 105, DK-2100 Copenhagen, Denmark

## Abstract

**Background:**

*Bacillus cereus, Bacillus anthracis *and *Bacillus thuringiensis *belong to the genetically close-knit *Bacillus cereus sensu lato *group, a family of rod-shaped Gram-positive bacteria. pAW63 is the first conjugative plasmid from the *B. cereus *group to be completely sequenced.

**Results:**

The 71,777 bp nucleotide sequence of pAW63 reveals a modular structure, including a 42 kb *tra *region encoding homologs of the Type IV secretion systems components VirB11, VirB4 and VirD4, as well as homologs of Gram-positive conjugation genes from *Enterococcus*, *Lactococcus*, *Listeria*, *Streptococcus *and *Staphylococcus *species. It also firmly establishes the existence of a common backbone between pAW63, pXO2 from *Bacillus anthracis *and pBT9727 from the pathogenic *Bacillus thuringiensis *serovar *konkukian *strain 97-27. The alignment of these three plasmids highlights the presence of well conserved segments, in contrast to distinct regions of high sequence plasticity. The study of their specific differences has provided a three-point reference framework that can be exploited to formulate solid hypotheses concerning the functionalities and the molecular evolution of these three closely related plasmids. This has provided insight into the chronology of their divergence, and led to the discovery of two Type II introns on pAW63, matching copies of the mobile element IS*231*L in different loci of pXO2 and pBT9727, and the identification on pXO2 of a 37 kb pathogenicity island (PAI) containing the anthrax capsule genes.

**Conclusion:**

The complete sequence determination of pAW63 has led to a functional map of the plasmid yielding insights into its conjugative apparatus, which includes T4SS-like components, as well as its resemblance to other large plasmids of Gram-positive bacteria. Of particular interest is the extensive homology shared between pAW63 and pXO2, the second virulence plasmid of *B. anthracis*, as well as pBT9727 from the pathogenic strain *B. thuringiensis *serovar *konkukian *strain 97-27.

## Background

The *Bacillus cereus sensu lato *family of rod-shaped Gram-positive bacteria contains six subspecies that are genetically very close [1] but nonetheless have highly specialized lifestyles, especially as concerns their respective virulence spectra. Most notable are *B. cereus sensu stricto*, an opportunistic pathogen which has been implicated in food poisoning [2] and endophtalmitis [3], *B. anthracis*, the etiological agent of anthrax [4], and *B. thuringiensis *which produces δ-endotoxin crystals that are toxic to insect larvae [5]. These subspecies are thought to have emerged from a common ancestor following a series of genetic rearrangements mediated *inter alia *by mobile DNA elements (transposons, insertion sequences and phages), in synergy with various mechanisms of horizontal gene transfer (conjugation, transduction or transformation), leading to the acquisition of virulence genes. This is exemplified by the presence of large virulence plasmids in *B. anthracis *(pXO1 and pXO2) [6], emetic strains of *B. cereus *[7] and *B. thuringiensis *that carry the genes responsible for the main phenotypic properties by which these bacteria can be distinguished.

While the inter- and intra-molecular movements of mobile elements can obviously have major consequences for the organization and composition of the host genome, it is probably the mechanism of conjugation that best enables the dispersion of these elements throughout the gene pool. Several conjugation systems have been described in Gram-negative bacteria, all of them involving the formation of a sex pilus to bring the participants in close contact, followed by the actual transfer of genetic material via a type IV secretion system (T4SS) [8]. There is much less data available concerning conjugation among Gram-positive bacteria, but the present consensus distinguishes four main transfer strategies, the most common of which seems to be that of the so-called broad host range plasmids. At present, the best characterized of these are the multiresistance plasmids pSK41 [9] and pGO1 [10] from *Staphylococcus*, pRE25 [11] from *Enterococcus*, pMRC01 [12] from *Lactococcus *and pIP501 [13] from *Streptococcus *as recently reviewed by Grohmann and coworkers [14].

The broad-host-range conjugative plasmid pAW63 was identified in *B. thuringiensis *serovar *kurstaki*, where it displays an efficient ability to conjugate in liquid medium, both for its own transmission (around 10^-3 ^transconjugants per donor in broth mating, up to a frequency of 1:1 between *kurstaki *strains) as well as that of small mobilizable plasmids [15]. Moreover, heterologous conjugation experiments have shown that it is also capable of transfer to its cousins *B. thuringiensis *serovar *israelensis *and *B. cereus*, as well as to *Bacillus sphaericus*, *Bacillus licheniformis *[15] and less closely related species such as *Listeria innocua *and *Enterococcus faecalis *(A. Wilcks, pers. comm.; G. Van der Auwera and J. Mahillon, unpublished).

In the present study, the complete sequence determination of pAW63 has led to a functional map of the plasmid yielding insights into its conjugative apparatus, which includes T4SS-like components, as well as its resemblance to other large plasmids of Gram-positive bacteria. Of particular interest is the extensive homology shared between pAW63 and pXO2, the second virulence plasmid of *B. anthracis *[16], as well as pBT9727 from the pathogenic strain *B. thuringiensis *serovar *konkukian *strain 97-27 [17].

## Results

### pAW63 is a 71,777 bp circular molecule

**Figure 1:**
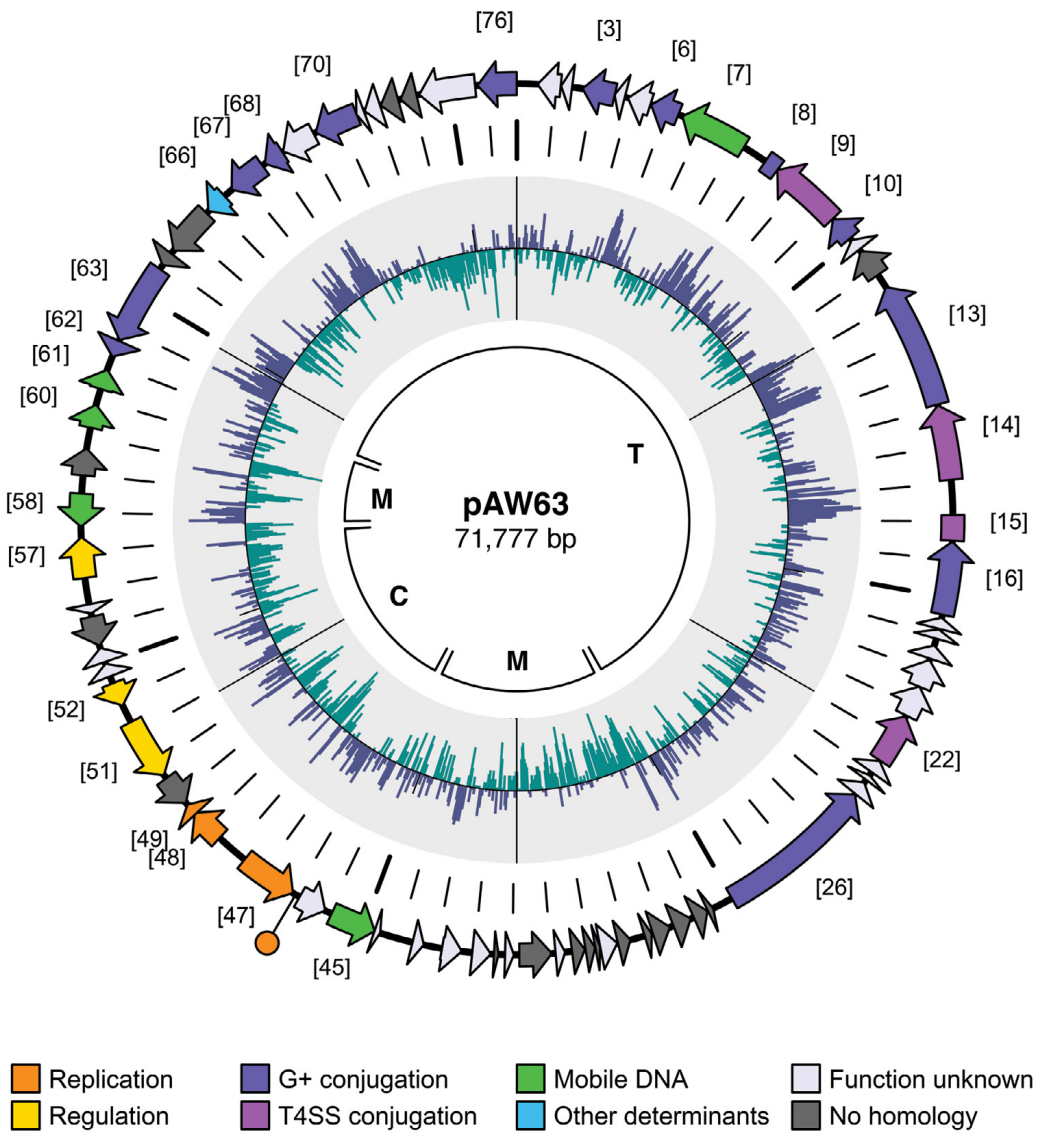
**Circular map of pAW63. **Coding Sequences (CDS) are represented by block arrows on the outer circle. Predicted functions/homologies are indicated by the color key featured below; numbers in brackets refer to main CDSs (see Table 1 for details). The first circle from the center delineates the functional modules identified on the plasmid; **T **indicates the transfer (*tra*) region, **C **indicates the control region (replication and regulation), and **M **indicates the two mobile DNA-associated flanking regions. The second circle from the center is a circular bar graph of the G+C composition percentage of the plasmid sequence, with the overall mean value (33.8%) as baseline; values above the line are G+C rich (max value 51% G+C) and values below the line are A+T rich (min value 16% G+C). The third circle from the center is a graduated size scale with small tick marks every 1 kb and large tick marks every 10 kb.

The complete nucleotide sequence of pAW63 was determined to be 71,777 bp long with a G+C content of 33.8%. Analysis of the coding content and organization revealed few intergenic regions, and a total of 76 coding sequences (CDSs) were identified (Fig. 1), of which all but 8 were found to be in the same orientation (defined as counter-clockwise). Interestingly, these 8 CDSs were all located in close proximity to each other, and one of these (CDS 47) corresponded to a component of the pAW63 replicon, which was isolated and sequenced in a previous study [18] while several others were later assigned putative functions as mobile genetic elements as detailed further in the text. The sequence was found to have an 81% coding ratio with an average CDS length of 768 bp. The largest CDS identified was CDS 26, weighing in at 4,248 bp. Several CDSs were associated to distinct spikes in G+C composition and will be discussed individually. The BLAST similarity searches showed that most of the 76 predicted CDSs encoded proteins with similarity to proteins from other organisms, although 36 of these were hypothetical proteins and only 26 could be attributed biological functions, while 14 of the predicted genes did not have any known homologs at all. The 26 genes encoding proteins with discernible functions were assigned to functional categories according to a classification scheme adapted from Riley [19]. The physical details and relevant BLAST hit results (scored by percentage of amino acid identity) of all 76 CDSs are summarized in Table 1.

**Table 1:** CDSs of pAW63: homologies and comparison with pXO2 and pBT9727

**pAW63**	**s**	**coordinates**		**size **(aa)	**most relevant homology**	**pXO2**		**pBT9727**	
CDS*		start	stop		function / *microorganism */ (amino acid identity percentage 'id %')	CDS*	id %	CDS*	id %
#001	-	1151	542	203	-	#001	77%	#001	67%
#002	-	1450	1162	96	hypothetical protein Efae03001129 *Enterococcus faecium *(29%)	#002	55%	#002	52%
#003	-	2585	1727	286	chromosome segregation ATPase *E. faecium *(25%)	#004	80%	#003	67%
#004	-	2857	2581	92	-	#005	92%	#004	62%
#005	-	3554	2945	203	-	#006	93%	#006	60%
#006	-	4333	3577	252	Tn*916 *ORF 14, similar to NLP/p60 family lipoprotein *E. faecalis *(37%)	#007	88%	#007	78%
#007	-	6254	4448	602	Type II intron reverse transcriptase IepA *Bacillus megaterium *(44%)	-	-	-	-
#008	-	7314	6927	129	Tn*916 *ORF 14, similar to NLP/p60 family lipoprotein *E. faecalis *(37%)	#007	77%	#007	61%
#009	-	9250	7315	645	VirB4, Type IV secretory pathway *E. faecium *(37%)	#008	95%	#008	80%
#010	-	9935	9266	223	ATPase involved in DNA repair *E. faecium *(30%)	#009	91%	#009	75%
#011	-	10315	10000	105	-	#010	95%	#010	79%
#012	-	11142	10377	255	-	-	-	-	-
#013	-	14900	11528	1124	adhesin AidA, Type V secretory pathway *E. faecium *(25%)	#013	74%	#013	48%
#014	-	16905	14925	660	VirD4, Type IV secretory pathway *E. faecium *(42%)	#014	79%	#014	85%
#015	-	18508	17839	223	VirD4, Type IV secretory pathway *E. faecium *(38%)	#015	94%	#014	83%
#016	-	20463	18534	643	putative membrane protein (no bacterial homologs)	#016	65%	#015	50%
#017	-	20847	20547	100	-	#017	72%	#016	43%
#018	-	21116	20864	84	-	#018	89%	#017	63%
#019	-	21662	21299	121	hypothetical protein *Listeria monocytogenes *(37%)	#020	83%	#018	58%
#020	-	22452	21702	250	conserved hypothetical protein*L. monocytogenes *(34%)	#021	99%	#019	70%
#021	-	23282	22448	278	conserved hypothetical protein*L. monocytogenes *(32%)	#022	69%	#020	61%
#022	-	24648	23316	444	VirB11, Type II/IV secretion system *L. monocytogenes *(35%)	#023	87%	#021	70%
#023	-	25024	24664	120	conserved hypothetical protein *L. monocytogenes *(37%)	#024	94%	#022	70%
#024	-	25273	25123	50	-	#025	57%	-	-
#025	-	25735	25360	125	-	#026	77%	#023	68%
#026	-	30002	25754	1416	cell surface protein, similar to Rhs family *Bacillus cereus *(20%)	#027	78%	#024	84%
#027	-	30709	30556	51	-	-	-	-	-
#028	-	31117	30724	131	-	-	-	-	-
#029	-	31693	31204	163	-	-	-	-	-
#030	-	32268	31794	158	-	-	-	-	-
#031	-	32492	32300	64	-	-	-	-	-
#032	-	33216	32880	112	-	-	-	-	-
#033	-	33820	33694	42	-	-	-	-	-
#034	-	33693	33234	153	-	-	-	#026	58%
#035	-	34082	33827	85	-	-	-	-	-
#036	-	34439	34100	113	-	-	-	-	-
#037	-	34874	34622	84	-	-	-	#028	66%
#038	-	35824	34972	284	-	-	-	-	-
#039	-	36199	35968	77	-	-	-	#033	50%
#040	-	36488	36371	39	-	-	-	-	-
#041	-	37060	36571	163	-	#031	88%	#034	39%
#042	-	37861	37336	175	-	#032	84%	#036	50%
#043	-	38649	38346	101	-	#033	90%	#037	60%
#044	-	39688	39532	52	-	#034	66%	-	-
#045	-	40902	39684	406	prophage helix-turn-helix protein *Bacillus cereus *G9241 (25%)	#036	89%	#038	59%
#046	-	41848	41089	253	-	#038	88%	#045	70%
#047	-	43592	42050	514	replication protein RepE *E. faecalis *(40%)	#039	96%	#046	83%
#048	+	44458	45385	309	replication-associated protein RepB *E. faecalis*	-	-	-	-
#049	+	45347	45671	108	putative replication-associated (no homologies) (31%)	-	-	-	-
#050	-	46557	45735	274	-	-	-	-	-
#051	-	48301	46663	546	pheromone binding protein *B. cereus *G9241 (73%)	#089	90%	#055	80%
#052	-	49424	48788	212	DNA-binding protein *B. cereus *G9241 (53%)	#093	61%	#059	77%
#053	+	49620	49968	116	conserved hypothetical protein *Staphylococcus aureus *(31%)	#095	75%	#062	71%
#054	+	50020	50407	129	group specific protein *B. cereus *ZK (75%)	#096	77%	#063	76%
#055	-	51260	50471	263	hypothetical protein lpl1076 *Legionella pneumophila *(49%)	-	-	-	-
#056	-	51589	51343	82	hypothetical protein *B. cereus *ATCC 14579 (60%)	-	86%	-	-
#057	+	52288	53362	358	RapD response regulator *B. thuringiensis *(94%)	-	-	-	-
#058	-	54506	53636	290	transposase, IS5 family *Aneurinibacillus thermoaerophilus *(52%)	-	-	-	-
#059	+	54988	55699	237	-	-	-	-	-
#060	+	56252	56870	206	site-specific recombinase, resolvase *Clostridium thermocellum *(54%)	-	-	#065	69%
#061	+	57303	57834	177	phage site-specific recombinase *B. cereus *ATCC 14579 (64%)	#101	-	#066	73%
#062	-	58650	58203	149	CAAX N-terminal protease *B. cereus *ATCC 14579 (35%)	#103	-	#071	68%
#063	-	60821	58679	714	DNA topoisomerase, TrsI/TraI *Lactococcus lactis *(45%)	#104	-	#072	74%
#064	-	61323	60942	127	-	-	-	-	-
#065	-	62731	61429	434	conserved hypothetical protein *B. cereus *G9241 (35%)	-	-	-	-
#066	-	63383	62798	195	AbiQ abortive infection mechanism *L. lactis *(34%)	-	-	#073	28%
#067	-	64659	63633	342	signal transduction histidine kinase *E. faecium *(38%)	#107	90%	#074	83%
#068	-	65290	64738	184	putative membrane-bound hydrolase *Bacillus licheniformis *(32%)	#109	-	#075	92%
#069	-	66240	65313	309	-	#110	83%	#076	60%
#070	-	67475	66302	391	ATPase involved in DNA repair *E. faecium *(42%)	#111	77%	#077	94%
#071	-	67681	67471	70	-	#112	62%	#078	75%
#072	-	68132	67703	143	-	#113	80%	#079	69%
#073	-	68628	68151	159	-	-	-	-	-
#074	-	69124	68695	143	-	-	-	-	-
#075	-	70679	69197	494	-	-	49%	#079	50%
#076	-	71773	70744	343	ATPase involved in DNA repair *E. faecium *(36%)	-	96%	#080	83%

* CDS numbers are abbreviated forms originating from their formal designations: pAW63-###, GBAA_pXO2_0###, pBT9727_0###

### pAW63 generic relationships

Most of the significant similarity results originated from Gram-positive bacteria species such as *Enterococcus *spp., *Streptococcus *spp., *Staphylococcus *spp., *Lactococcus *spp. and *Listeria *spp. in addition to *Bacillus halodurans*, *Bacillus subtilis*, *Bacillus licheniformis *and the members of the *B. cereus sensu lato *group. It is of particular interest to note that out of the 76 CDSs predicted on pAW63, 50 CDSs showed strong similarity (between 48.8 and 98.7% amino acid identity, with an average of 81.1%) to CDSs found on pXO2 from *B. anthracis *[GenBank:NC_007323] and 49 CDSs showed strong similarity (between 43.8 and 97.4% amino acid identity, with an average of 70.5%) to pBT9727 from *B. thuringiensis *serovar *konkukian *[GenBank: CP_000047], with 42/76 (55.3%) of these CDSs being shared by all three plasmids. This was consistent with previous observations of sequence similarities between these plasmids [18,20,21].

### Replication and regulatory functions are grouped in an 8 kb 'control center'

All the elements that were putatively identified as being directly involved in plasmid replication, copy control or other regulatory processes were found to reside within an 8 kb region of the plasmid containing 12 CDSs (CDS 46 to 57), of which 6 could not be assigned to a functional category. This region was delineated by mobile genetic elements or remnants thereof, and contained five of the eight CDSs that were found in clockwise orientation, the other three being located in its gene mobility-associated flanking sequences.

The 4.1 kb replicon of pAW63 was characterized in a previous study [18] and classified as belonging to the pAMβ1 family of theta-replicating conjugative plasmids, with which it shares a similar *cis*-functioning origin of replication (*ori*). The largest of the four CDSs contained in this region, Rep63A (CDS 47), displayed strong similarity to the replication proteins of several plasmids in this family, as well as 96% amino acid identity with the RepS protein of the pXO2 replicon [18]. Rep63B, the second largest CDS of the replicon (CDS 48), displayed strong similarity with copy control proteins RepB and PrgP from the *Enterococcus faecalis *conjugative plasmids pAD1 [22] and pCF10 [23], respectively [18]. Although the two smaller CDSs (49 and 50) did not display any significant homologies, the authors noted that one of these, CDS 49, showed properties (location, size, orientation and hydrophilicity) that likened it to *repC *and *prgO*, which are putative genes encoding stability functions on pAD1 and pCF10, respectively.

Upon further sequencing of the plasmid, three additional putative regulatory elements were identified in close proximity to the replicon. These elements (CDS 51, 52 and 57) were homologs of genes that are highly conserved throughout the *B. cereus *group as well as in some less closely related species. CDS 51 and 52 displayed significant homologies to an oligopeptide ABC transporter functioning as a pheromone binding protein, and to a DNA-binding protein, respectively. The clockwise-oriented CDS 57 showed a distinctly low overall G+C content and was found to encode a protein with 94% identity to RapD, a response regulator aspartate phosphatase acting as a transcriptional activator involved in the regulation of sporulation [24].

### The conjugative functions of pAW63 are grouped in a 42 kb *tra *region

All 15 CDSs assigned putative conjugative functions were found to be located within a 42 kb region of the plasmid (CDS 1 to 26 and 62 to 76; hereafter referred to as the *tra *region). Every one of the 41 CDSs predicted within this region was in the same counter-clockwise orientation, suggesting an operon-style transcription scheme may be in place, which would be consistent with the genetic organization of most known conjugative systems [25].

#### pAW63 encodes three homologs of Gram-negative T4SS components

Three CDSs were found to encode proteins with significant similarity to components of the Vir secretion system, originally characterized in *Agrobacterium tumefaciens*, which is the archetypal model for the Type IV Secretion System (T4SS) that is believed to carry out the conjugative process in Gram-negative bacteria, as recently reviewed [8,26]. Briefly, the T4SS can be summarized as a two-step mechanism [27] which involves the replication of the plasmid DNA by effector proteins followed by the translocation of a single-stranded molecule across the cell envelope into the recipient cell. The transfer is powered by the action of a molecular pump and the DNA is thought to travel through a channel formed by a core protein complex, while the necessary close cell-to-cell contact is mediated by surface structures.

Analysis of the pAW63 sequence (Fig. 1) showed that CDSs 14 and 15 were in fact the two halves of an interrupted CDS encoding a homolog of the VirD4 component, which has been shown to function as a coupling protein between the DNA strand replication machinery and the transmembrane transfer complex and powerhouse. Interestingly, the 935 bp space between them was found to be completely devoid of coding sequences and showed a distinctly higher G+C content than was considered average for the rest of the plasmid. CDS 9 displayed 37% identity with VirB4, a membrane associated N-triphosphatase which is thought to play a role in providing the energy needed to power the actual DNA transfer. CDS 22 displayed 35% identity to VirB11, a subunit implicated in both Type II and Type IV secretion systems that was shown to be necessary for conjugation, although its exact role remains unclear. Structural analyses have shown it to possess a transmembrane domain as well as an ATPase domain, and recent research suggests that the subunits are arranged as a dynamic hexameric assembly associated with the inner membrane and functioning as a gating component [28].

#### pAW63 encodes homologs to components of Gram-positive conjugation systems from diverse species

Another 10 CDSs were classified as conjugative on the basis of their homologies, this time to genes from Gram-positive conjugation systems. Most notably, CDS 63 showed a distinctly high G+C content and was found to encode a topoisomerase displaying 45% identity to the TrsI/TraI protein from the *Lactococcus lactis *conjugative plasmid pMRC01 [12]. This component of the lactococcal conjugative system is thought to function as a relaxase, a role equivalent to that of VirD2 in the T4SS machinery.

CDSs 67 and 70 both displayed significant homology to LtrC-like (from the Lactococcal transfer ORF C) putative conjugative elements from *Streptococcus*, *Staphylococcus*, *Lactococcus *and *Listeria *species (with an average 30% identity), although they had different 'most relevant BLAST hits', as shown in Table 1. The function of the LtrC element itself, which was identified in the *tra *region of the lactococcal conjugative plasmid pRS01 [29], has not yet been determined.

CDSs 6 and 8 were predicted to be the two halves of an interrupted protein with significant resemblance to ORF 14 from the enterococcal conjugative transposon Tn*916*, a self-transmissible molecule encoding tetracycline resistance [30]. The function of Tn*916 *CDS 14 has not yet been elucidated, but further results of the similarity search displaying comparable identity levels included a soluble lytic murein transglycosylase from *E. faecium *as well as the invasion-associated extracellular protein Iap from *L. monocytogenes*.

Furthermore, three CDSs (CDS 13, 16 and 26) were predicted to encode proteins that may be involved in the establishment of intimate cell-to-cell contact, either as aggregation substances secreted into the medium, or as cell surface determinants. CDS 13 showed a distinctly high G+C content in its second half, which contained two tracts of distinct repeated units that were predicted to correspond to a series of helix-turn-helix motifs followed by a series of pleated sheets in the protein product. It also displayed 25% identity with the adhesin AidA from the Type V secretory pathway of *E. faecium*, which relies on an autotransporter system for cytolysin secretion. The adhesin itself is primarily implicated in the pheromone-mediated enterococcal mating process as an aggregation substance, but it has also been shown to act as an adhesion factor in the course of infection of eukaryotic cells by pathogenic *E. faecalis *strains and as such it may be considered a virulence determinant [31]. CDS 26 was the largest CDS found on the plasmid (4.2 kb) and showed little significant resemblance to any known sequences except for a positive match (20% identity) with a 3.3 kb long cell-surface protein from the *B. cereus *reference strain ATCC14579 and another (23% identity) with a 2.37 kb long Rhs family protein (for Recombination hot spot) from *Bifidobacterium longum*. Rhs products are thought to possess properties typical of cell surface proteins and often present internal rearrangements that lead to antigenic variation [32]. CDS 16 was unlike any known bacterial sequences (apart from those of plasmids pBT9727 and pXO2) but the putative 643 residue protein was predicted to have a secondary structure consisting essentially of hydrophilic helices, suggesting it may function as a surface-associated or free-acting aggregation substance.

Finally, the remaining CDSs correspond to ATPases predicted to be involved in segregation and DNA repair (CDSs 3, 10, 62 and 76), as well as a predicted membrane-bound hydrolase (CDS 68).

### pAW63 and mobile genetic elements

Five CDSs were readily identified as mobile genetic elements on the basis of their homologies. Standing apart from the other four by both character and location, CDS 7 was found positioned right between the two fragments of the interrupted Tn*916 *ORF 14-like element and displaying 44% identity to IepA, a group II intron-encoded protein from *Bacillus megaterium *which further BLAST results revealed to be a reverse transcriptase and maturase. This immediately led to the conclusion that CDS 7 was part of a group II intron that had inserted itself in the uninterrupted ancestor of CDSs 6 and 8, a finding supported by the skewed G+C content profile corresponding to the site of the proposed insertion. The element was tentatively named B.th.I1 (for *B. thuringiensis *Intron #1). Furthermore, a closer examination of the 935 bp fragment of seemingly non-coding DNA with high G+C content found interrupting the VirD4 homolog (CDSs 14 and 15) revealed that the extremities of this insert were nearly identical to those of the B.th.I1 intron mentioned above, indicating the presence in this locus of a second retroelement presumably derived from the first. This second element was tentatively named B.th.I2 (for *B. thuringiensis *Intron #2).

Three other of these CDSs (CDS 58, 60 and 61) were found in close proximity to each other by the regulation side of the proposed 'control center' region, as mentioned previously, and were predicted to encode various types of DNA recombinases.

Finally, CDS 45 was found flanking the replication side of the replication control center and showing similarity to a prophage helix-turn-helix protein from the *B. cereus *strains G9241 and ATCC 14579. The 10 kb region downstream of CDS 45 was found to encode 18 rather short CDSs (≈300 bp on average) with no significant homologies in a configuration reminiscent of typical bacteriophage gene structure, which may correspond to a prophage integrated within the plasmid sequence. Intriguingly, while the first 4 kb of this region showed a G+C content in accordance with the rest of the plasmid, the following 6 kb segment showed a distinctly low G+C content along most of its length.

As a counterpoint to the considerable amount of phage-derived genetic material tentatively identified on the plasmid, CDS 66 displayed significant similarity to AbiQ, a single-protein abortive infection mechanism from *L. lactis *which limits phage dissemination by shutting down the lytic cycle and leading the infected host cells to their death [33].

### pAW63 shares a common backbone with pXO2 from *B. anthracis *and pBT9727 from *B. thuringiensis konkukian*

Similarities between pAW63 and the virulence plasmid pXO2 from *B. anthracis *had been observed previously [18,20] and further investigation (C. Kuske, pers. comm.) had yielded thirty short sequences (400 bp long on average) corresponding to regions of pAW63 which had been shown to hybridize to pXO2. This data was used in this study as the basis for the sequencing of pAW63, in a strategy that involved the outlining of a backbone sequence from which primers pairs were designed to amplify and individually sequence the intervening regions.

**Figure 2:**
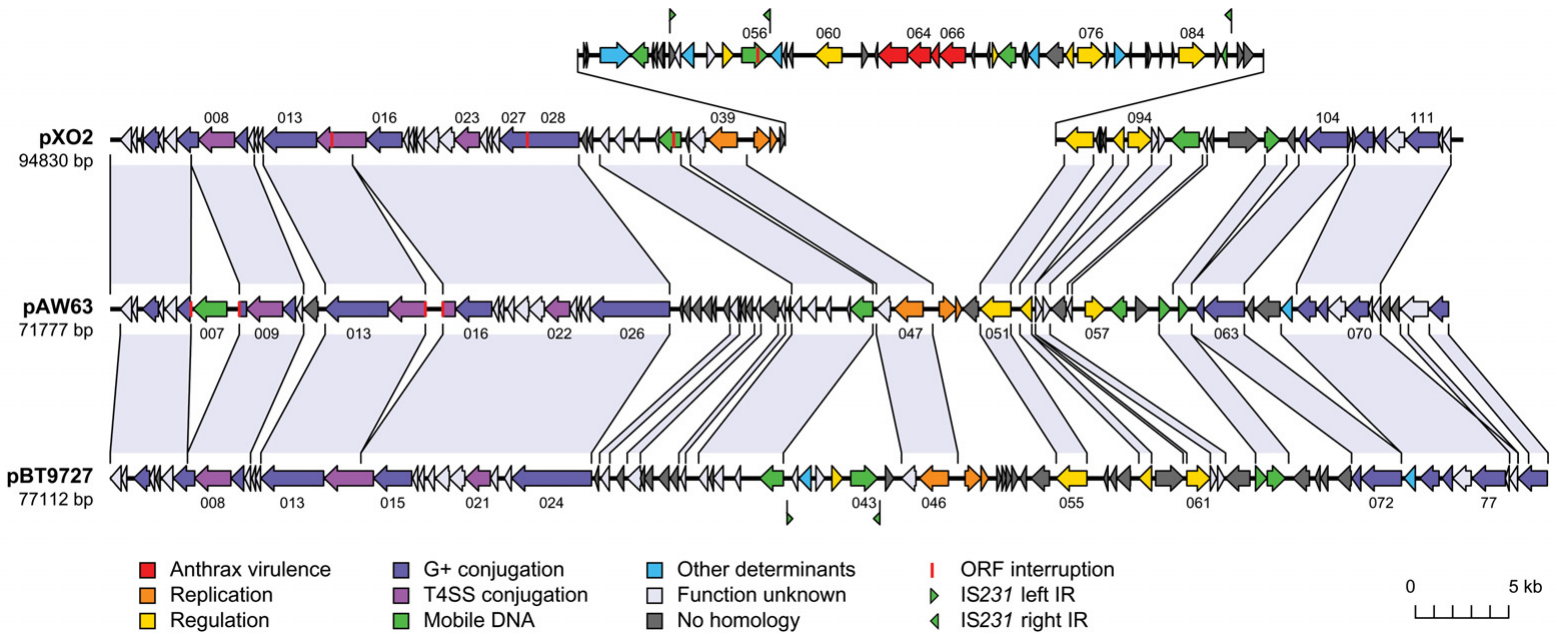
**Linear alignment of pXO2, pAW63 and pBT9727. **CDSs are represented by block arrows. Several CDS numbers (see Table 1) are indicated for reference on each plasmid, just above or below their representation. Predicted functions/homologies are indicated by the color key featured below. Well conserved segments of the plasmids are paired by shaded regions (>40% amino acid identity); percentages for specific CDS pairs can be found in Tables 1 and 2. The proposed PAI of pXO2 is raised above the rest of the sequence for clarity. Scale is indicated by the bar in the lower right-hand corner.

These premises had guaranteed at least several hits to the virulence plasmid from *B. anthracis*, pXO2, and the final sequence of pAW63 certainly did not disappoint. Indeed, a preliminary alignment of their sequences showed that aside from a 37-kb region found only on pXO2 corresponding to the region containing the anthrax capsule genes and associated regulatory elements, they possessed a highly similar genetic makeup. Furthermore, the BLAST results had also revealed the existence of a third closely related plasmid, pBT9727, which was recently published along with the genome sequence of its host strain *B. thuringiensis *serovar *konkukian *strain 97-27 [GenBank:NC_005957] and shows a comparable overall gene organization and coding content to that of the other two. These findings were consolidated by performing a BLAST Score Ratio Analysis of the predicted proteomes of the three plasmids, the results of which indicated a high level of synteny, with the pAW63 and pBT9727 pair showing the highest ratio of shared CDSs although the associated identity percentages were the poorest. Regarding the CDSs shared by each of these plasmids with pXO2, pBT9727 possessed the highest number of these but for the most part pAW63 showed higher identity percentages (Tables 1 and 2). A global phylogenetic analysis was performed on the three proteomes by systematically aligning the 42 homologous predicted proteins shared by all three plasmids together along with their most relevant common BLAST hit result and building a tree from the four sequences (data not shown). Results were consistent for all quartets examined and suggested that pBT9727 had been the first to branch off from the common ancestor, while pAW63 and pXO2 diverged later. The detailed alignment of the three sequences shown in Figure 2 clearly illustrates the extent of the synteny observed between these plasmids.

**Table 2:** Comparison of pXO2 and pBT9727 CDSs with no homologs on pAW63

***B. anthracis *CDS**	**id %**	***B.t. konkukian *CDS**	**Putative gene functions**
GBAA_pXO2_0011	65%	pBT9727_0011	-
GBAA_pXO2_0012	60%	pBT9727_0012	-
GBAA_pXO2_0028	67%	pBT9727_0024	-
GBAA_pXO2_0035	64%	pBT9727_0038	-
GBAA_pXO2_0040	91%	pBT9727_0047	replication-associated protein
GBAA_pXO2_0041	79%	pBT9727_0048	replication-associated protein
GBAA_pXO2_0052	89%	pBT9727_0039	-
GBAA_pXO2_0053	72%	pBT9727_0040	CAAX N-terminal protease
GBAA_pXO2_0054	92%	pBT9727_0041	-
GBAA_pXO2_0055	97%	pBT9727_0042	transcriptional regulator TetR
GBAA_pXO2_0056	96%	pBT9727_0043	IS*231 *transposase
GBAA_pXO2_0057	66%	genomic?	bacitracin transport permease
GBAA_pXO2_0061	39%	genomic?	-
GBAA_pXO2_0075	36%	genomic?	sensor histidine kinase
GBAA_pXO2_0086	81%	pBT9727_0043	IS*231 *transposase
GBAA_pXO2_0094	93%	pBT9727_0061	DNA-damage repair protein

### The *tra *region of pAW63 finds its equivalent on both pXO2 and pBT9727, with high levels of identity and contrasting discrete variations

Both pXO2 and pBT9727 were found to possess an approximately 42 kb long region that was almost identical to the *tra *region of pAW63 with respect to gene structure and organization, as illustrated in Fig. 2. All of the CDSs identified on pAW63 as possible conjugative genes were found to have highly similar homologs on both of these plasmids (see percentages in Tables 1 and 2), although several displayed key differences as detailed below.

While the copy of the Tn*916 *CDS 14-like element present on pAW63 had been found to be encoded by two CDSs (CDSs 6 and 8) due to the insertion of the group II intron B.th.I1 (see above), the corresponding homologs of this element were uninterrupted on pBT9727 and pXO2. The sequence comparison of these 'native' versions of the gene with the one containing the intron allowed for the precise site of insertion to be determined. This region of the Tn*916 *CDS 14-like element was then aligned with its closest homologs as identified by BLAST search, revealing that the insertion had taken place in a relatively well-conserved domain of the gene. This is consistent with the observation that group II introns target specific genes or gene domains [34] despite the lack of experimental data available concerning the insertion of this particular element.

Similarly, the three plasmids possessed separate versions of the VirD4 homolog. The CDS encoding this gene was found to be interrupted in a distinct manner on both pXO2 and pAW63, while it appeared to be intact on pBT9727. As indicated previously, the pAW63-borne homolog (CDS 14 and 15) was interrupted by the putative ORF-less group II intron B.th.I2, while the cause of the disruption of the pXO2-borne homolog was a frameshift due to the deletion of two nucleotides in a C-rich stretch located at two-thirds of the original protein.

Likewise, homologs for the putative cell-surface protein encoded by CDS 26 of pAW63 were found on both pXO2 and pBT972, albeit in two different forms. While pBT9727 was found to possess a presumably intact version of the gene, the pXO2-borne version appeared to be disrupted by a single nucleotide deletion causing a frameshift two-thirds into the original protein. This was confirmed to be a genuine feature and not a sequencing artifact by comparing the three available sequences of pXO2 [GenBank:NC_002146, GenBank:NC_007323, GenBank:NC_003981] which had identical frameshifts in the corresponding locus.

Finally, the three homologs of the putative adhesin (CDS 13 on pAW63) were found to differ in a very interesting way. While the first half of the protein was well conserved in all three versions, the second half containing the repeated motifs was almost identical between pXO2 and pAW63, although pXO2 lacked several repetitions of the basic units, and pBT9727 possessed completely different basic units which nevertheless gave rise to highly similar secondary structure predictions.

### Sequence variability is highest in the replication control center area and is associated with the presence of multiple mobile genetic elements

The mobile DNA-based borders of the replication control center of each plasmid were found to be comparable as follows. The putative prophage structure bordering the replicon (CDS 27 to 45 on pAW63) was found on all three plasmids, although the second part of this structure was poorly conserved, to the point of being absent from pXO2. The other side of this region was delineated in all three cases by variants of the site-specific recombinase found on pAW63 (CDS 61). The area between the replicon and this recombinase was highly variable between the three plasmids, with pAW63 featuring the most CDSs therein (CDS 50 to 60).

Regarding the regulatory elements present in this region, it was observed that while both the putative pheromone receptor (CDS 51) and the DNA-binding protein (CDS 52) identified on pAW63 were also present as close homologs on the two other plasmids, the putative transcriptional activator RapD (CDS 57) was not found in any form on either of them. On the other hand, both pBT9727 and pXO2 were found to possess almost identical copies of two other genes that were not present on pAW63: a homolog of the DNA-damage repair gene *uvr *from *B. subtilis*, and the gene encoding the transcriptional activator TetR.

### A recent recombination event caused pAW63 to exchange part of its replicon

**Figure 3:**
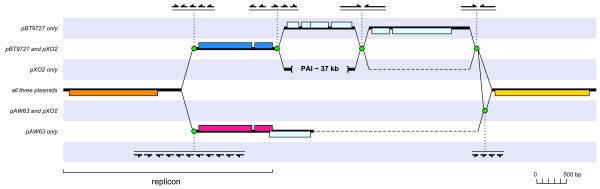
**Relational diagram of the replicon region of the three plasmids. **Comparison of the replicon region of each plasmid reveals a complex structure of palindromic and/or iterative elements serving as node points for recombination events. Sequence segments are represented by thick horizontal lines joined by solid diagonal lines. The background striping highlights 'shared' *versus *'unique' sequence segments as indicated by the text legend on the left hand side. Horizontal dashes are spacers to indicate a shorter length or lack of corresponding segment. Node points putatively involved in recombination events are represented by green circles; iterative and/or palindromic sequence units by half arrows; CDSs by rectangles, above the sequence line to indicate clockwise orientation, or below to indicate counter- clockwise orientation.

It is interesting to note that while the origin of replication and main replication protein Rep63A (CDS 47) were found to be highly conserved across all three plasmids, no homologs were found for the two other replication-associated CDSs of pAW63 (CDS 48 and 49), but two CDSs of similar size and orientation were found in equivalent locations relative to the common backbone on each of the other two plasmids, suggesting they share a functional homology with the two pAW63 CDSs, despite their lack of sequence similarity. Furthermore, these pBT9727- and pXO2-borne pairs of CDSs were found to share a high level of sequence identity with each other, suggesting that the original corresponding part of the pAW63 replicon had been exchanged for its present form at some point following the divergence of pXO2 and pAW63. In support of this idea, a similar pattern of resemblance/difference was observed for a series of partially palindromic iterons present in the vicinity of the replication genes which were previously believed to be implicated in the replication process. While the iteron sequences found on pBT9727 and pXO2 were by no means identical, they did share similar repeated motifs that were completely different from those identified on pAW63. The discovery of further repetitions of these various motifs as well as several additional palindromes in key locations of the sequence have led to the elucidation of a complex structure of palindromic and/or iterative elements serving as node points for recombination events that may have been responsible for major divergences in synteny between the three plasmids in the replicon area, as illustrated in Figure 3.

### Comparative evidence supports the existence of a 37 kb pathogenicity island (PAI) on pXO2

The 37 kb region of DNA located after the replicon on pXO2 was found to exhibit most of the characteristics of a bacterial pathogenicity island (PAI) as defined by Hacker and Kaper [35]. All of the genes carried by pXO2 that are known to play a capital role in the course of anthrax infection, such as the capsule genes, were clustered in this region, along with the regulatory genes *acpA *and *acpB*, which are directly associated with their expression during the development of the pathology [36]. Furthermore, this region had distinctive genetic properties from the rest of the plasmid, such as a lower G+C content (30.9% against 34.3%), and more variation in the orientation and phase of coding sequences. It was also found to be rich in mobile genetic elements (some cryptic or defective), such as multiple inactivated copies of the IS*231 *transposase [16,37] and a vestigial class II transposon.

The comparison of the sequences flanking the proposed PAI on pXO2 and their equivalents on pAW63 and pBT9727, as described above (Fig. 3), revealed the existence of specific nodes consisting of iterative and/or palindromic sequences that could be held responsible for the acquisition of the PAI region through a past recombination event, though not excluding the possibility of later rearrangements having taken place within this region.

### pXO2 and pBT9727 possess copies of Insertion Sequence IS*231*L in different loci, causing a visible interruption in synteny

The results of the three-way BLAST Score Ratio Analysis of this complex plasmid trio highlighted a group of five CDSs that were not present on the pAW63 sequence itself but were shared with a high degree of similarity between pBT9727 and pXO2. Their location was different on either plasmid with respect to the common backbone, thus representing a significant interruption in the synteny of the plasmids. It appeared that this small group had been previously described on pXO2 as an IS*231*-derived [38] Insertion Sequence (IS) and designated I*S231*L [37], although it was assumed to be incapable of autonomous transposition due to a frameshift in the CDS encoding the transposase. However, the copy of the I*S231*L element harboured by pBT9727 was found to have an uninterrupted and therefore supposedly functional transposase, as well as nearly perfect Inverted Repeats (IR), the canonical 20 bp boundaries of this type of mobile element matching those of the pXO2-borne IS*231*L element.

Interestingly, the copy of I*S231*L found on pBT9727 was found to be nested within the Tn*1546*-related [39] left IR of a novel Class II transposon belonging to the Tn*3 *family, encoding a single cryptic CDS and tentatively named Tn*Bt9727*. Likewise, the pXO2-borne copy of I*S231*L was nested within the Tn*ARS1*-related [GenBank AY780525] right IR of a vestigial class II transposon. No corresponding left IR was found for this element, but an IS*3 *family transposase was found in close proximity to the putative right IR, suggesting a possible structural association.

Furthermore, the presence of two other CDSs with homology to the IS*231 *transposase in the PAI of pXO2 prompted a search for other instances of IRs matching those of IS*231*L in the vicinity of these CDSs. A well-conserved copy of the right IR was indeed discovered near the end of the PAI beside a second CDS encoding an IS*231 *transposase-like gene (Fig. 2).

## Discussion

A large proportion of the pAW63 sequence was observed to possess significant similarity to plasmids originating from several different bacterial species harbouring fundamentally different conjugative and replication systems. The resulting hybrid combination of genes probably opens up the mating scope of the plasmid. The physical arrangement of the plasmid was identified as a composite structure consisting of a 42 kb *tra *region encoding conjugation functions, organised in typical operon fashion, and an 8 kb control center encoding the replication and regulation functions, joined by mobile genetic elements or remains thereof (Fig. 1).

Analysis of the replication control center of pAW63 revealed that it possessed copy control proteins similar to those found on the enterococcal conjugative pheromone plasmids pAD1 and pCF10. However, pAW63 conjugative transfer among *B. thuringiensis *strains has been shown to be independent from recipient-encoded pheromones [15]. It has been suggested that in addition to their role in the transfer process, host-produced pheromones may play a role in the replication of the pAD1 and pCF10 plasmids [23]. Given a similar copy control mechanism, the apparent independence of pAW63 from pheromone signalling may account for the greater host range of pAW63 compared to pAD1 and pCF10, by removing the requirement for a compatible pheromone system in the recipient mating partner. It was therefore very interesting to find near the pAW63 replicon two CDSs respectively encoding an ABC transporter-type pheromone receptor, homologous to those found in *Enterococcus *and *Listeria *species, and a close homolog of RapD, a transcriptional activator from *B. thuringiensis *serovar *morrisoni*. At this point it is unclear whether the function of these elements is restricted to either the plasmid copy control or the regulation of conjugation, or whether these processes are directly linked by common regulatory pathway components. Nonetheless, the presence of a putative pheromone-sensing mechanism on pAW63 suggests that the conjugative transfer process of the plasmid may follow alternate or optionally additive regulatory pathways depending on the species of the mating cells.

Regarding basic conjugative function, the presence on the plasmid of genes related to the T4SS system corroborates similar findings [40] concerning the transfer system of the streptococcal plasmid pIP501, which bears a *tra *region containing VirD4, VirB4 and Vir B11 homologs. Interestingly, all three of the pAW63 and pIP501 T4SS-like proteins correspond to components of the system that operate within the cell or in association with the inner membrane to prepare the plasmid DNA for transfer and provide motor function, and no homologs have been found on the plasmid to the 'outer' components. This could be explained by the idea that the inner part of the Gram-positive cell wall structure is sufficiently similar to that of the Gram-negative hosts for some of the 'inner' components, designated as effector and transporter molecules, of both transfer systems to share their main characteristics and possibly a common evolutionary history derived from replication mechanisms. Conversely, it seems reasonable to assume that major differences in the cell wall structure and composition, such as the presence of a thick peptidoglycan layer and the absence of an outer membrane, would be mirrored by the presence of specific protein components responsible for the 'external' steps of the conjugative process. This includes sensing of recipients, cell to cell attachment and the bridging of cell walls to effect DNA transfer, and requires the assembly of a core complex as well as cell surface structures. With respect to the latter, several putative cell surface- or membrane-associated proteins, such as the adhesin encoded by CDS 13 and the very large protein encoded by CDS 26, have already been identified in the *tra *region of pAW63, although further studies will be necessary to work out their specific roles in the conjugative process.

The presence of introns B.th.I1 and B.th.I2 within two genes of which at least one (the VirD4 homolog) may be considered essential to the conjugative process, poses the question of the functionality of the interrupted genes, as well as that of possible collateral effects caused by these elements. Regarding maintenance of functionality of the host genes, current models of group II intron splicing as well as experimental research show that intron excision during transcription leads to the production of a complete and functional protein [41]. In addition, this type of mobile element is thought to insert preferentially into certain conjugative genes such as relaxases, and it has been suggested that such an insertion actually enhances the efficiency of the conjugative system [34,42]. Furthermore, the relationship between B.th.I1, which encodes its own reverse transcriptase, and B.th.I2, which does not, deserves further investigation. While such a configuration has rarely been observed among eubacteria, ORF-less introns that were closely related to an intron encoding a reverse transcriptase located in the same genome have been identified in cyanobacteria and archeae [43]. It has been proposed that these CDS-less elements were derivatives of their 'complete' RT-encoding relatives [44]. Significantly, several of these 'degenerate' elements were shown to be mobile, raising the possibility that mobility may be supplied in *trans *[41] as is the case for the Mobile Insertion Cassette MIC231 trans-activated by IS*231 *transposase [37,45].

The comparison of the *tra *regions of pAW63, pBT9727 and pXO2 has provided several valuable indications on the relative importance of specific genes in the conjugative process. While pAW63 is known to be fully functional and quite efficient as a conjugative plasmid, pXO2 is believed to be incapable of autonomous transfer although it has been shown to be mobilizable by the conjugative plasmid pXO14 from *B. thuringiensis toumanoffi *[46], suggesting that pXO2 carries a set of conjugative genes that is incomplete or disrupted in one or several key components. Both pXO2 and the newly sequenced plasmid pBT9727 were now shown to possess a set of potential conjugation genes closely related to those of pAW63. The transfer capabilities of pBT9727 have not yet been assayed, but its sequence features point towards partial conjugative function at the very least, and possibly full self-transmissibility. For these reasons, further studies will focus on loci exhibiting discrete variations in order to expose their specific contribution to the transfer process. For instance, the unique interruptions found in the pXO2 homolog of the VirD4 element and in that of the putative cell surface protein encoded by CDS 26 on pAW63 may prove to be particularly significant, especially if pBT9727 is shown to be fully conjugative.

From a broader point of view, the combination of the phylogenetic analysis of the three proteomes with the elucidation of the structural features delineating the major sequence differences in the replication control center has made it possible to roughly reconstruct the molecular evolution of the plasmid trio. The revelation that pBT9727 was the first to diverge from the common stem, leaving pXO2 and pAW63 to branch off from an intermediate form some time later, carries several important implications for the ecology of the *B. cereus s.l. *family. At a very basic level, it highlights the mixed lineage of these plasmids, as attested by their distinct genetic properties (conjugation *versus *virulence) contrasting with the nearly perfect conservation on all three of the minireplicon of pXO2 [47]; plasmids which were isolated from strains that are treated quite differently, namely as a biopesticide *versus *a dangerous pathogen. This is particularly relevant in the light of recent incidents linking *B. cereus sensu stricto *strains to cases of anthrax-like pathologies [21,48,49], as well as the discovery that the cereulide genetic determinants of emetic *B. cereus *are located on a plasmid [7,50]. Most significantly, it makes a strong case for the pXO2 capsule genes region to be officially designated a pathogenicity island, a hypothesis further validated by the structural features typical of mature PAIs that were identified in this study. The recognition of pXO2 as a PAI-equipped virulence plasmid would also be consistent with the observation that most of the major known pathogens possess such PAIs, many of these carried by virulence plasmids, and it would put pXO2 on par with pXO1, which is known to carry its own anthrax toxin-bearing PAI. In addition, it should be noted that the configuration of IS*231*-related IRs observed on pXO2 (Fig. 2), which is quite likely due to the insertion of the IS*231*L element into the PAI after its acquisition by pXO2, entails that a region representing almost the entire PAI, containing all the capsule genes and their associated regulatory elements, can technically be considered an IS*231*-derived Mobile Insertion Cassette (MIC). As such, this cassette could undergo a transposition event provided a functional IS*231 *transposase is supplied in *trans*. Incidentally, this opportunity for virulence genes to be directly mobilized by a mobile genetic element from within a larger PAI is paralleled in pXO1 by the presence in its PAI of a Class II transposon, Tn*XO1*, which carries the anthrax germination operon *gerX *as its passenger genes [51].

## Methods

### Bacterial isolates and PCR amplification

*B. thuringiensis *strain HD73 containing pAW63 was grown in liquid LB medium at 30°C. 1 ml cultures grown overnight were centrifuged into a pellet, washed twice in ddH_2_0 and resuspended in 250 μl ddH_2_0. Freshly prepared aliquots of 5 μl of resuspended cell solution were used as template in each subsequent 50 μl PCR reaction. PCR amplification was performed using 'Hi-Fi polymerase mix' from Fermentas according to the manufacturer's specifications. For target fragments of over 3 kb it was necessary to adjust the MgCl_2 _concentration of the reaction mix as well as adding dimethylsulfoxide (DMSO) to optimize yield and specificity. Further details can be obtained upon request. Cloning procedures made use of One Shot^® ^TOP10 Electrocomp™ *E. coli *from Invitrogen, and transformation was performed according to the manufacturer's specifications.

### Sequencing strategy

The sequencing strategy took advantage of the previously observed similarity between pAW63 and the virulence plasmid pXO2 from *B. anthracis *[18,20]. Further investigation (C. Kuske, pers. comm.) yielded thirty short sequences (400 bp long on average) corresponding to regions of pAW63 which had been shown to hybridize to pXO2. This data was used to construct a backbone sequence on the basis of which primer pairs could be designed to amplify the intervening regions. The resulting amplicons were purified on gel using Qiagen QiaQuick Gel Purification Kit according to the manufacturer's specifications. Amplicons smaller than 5 kb were directly sequenced from the purified PCR sample, those between 5 and 10 kb were cloned into sequencing vectors, and those larger than 10 kb were each subcloned into separate libraries. All cloning procedures were performed using the Topo XL PCR cloning kit of Invitrogen according to the manufacturer's specifications. Amplicons were individually sequenced at the Flanders Interuniversity Institute for Biotechnology (VIB) Genetic Service Facility by double strand primer walking following standard procedures with a final accuracy of 99.98% per amplicon. The primary assembly of individual amplicon sequences was performed using the Seqman software package. The overall assembly quality was improved by sequencing regions of minimum 500 bp in length overlapping the extremities of adjoining amplicons using the same protocol as described above.

### Sequence analysis and annotation

The full assembly of the amplicons, overlapping fragments and the previously published pAW63 replicon was done manually using the Accelrys DS Gene sequence editor. The coordinate start of the sequence was assigned with respect to that of pXO2 in order to facilitate future comparative analyses between the two plasmids, for which the *B. anthracis *'Ames Ancestor' strain sequence of pXO2 was used [GenBank:NC_007323]. Potential coding regions were predicted with the prokaryotic gene finder EasyGene [52] using a hidden Markov model (HMM) pre-trained on the *B. anthracis *genome. Sequence similarity searches, alignments and further annotation were performed using the BioPerl open software package with standard default BLAST X and ClustalW parameters (details available on request). The BLAST score ratio analysis (BSRA) was performed as described by Rasko *et al. *[53]. Protein analyses were done using Accelrys DS Gene. Proteome phylogeny analyses were performed using the Tree Building Method (Neighbor Joining, uncorrected) in Accelrys DS Gene. All figures included in this work were generated using the BioPython, GenomeDiagram and ReportLab open software packages. The complete sequence of pAW63 has been deposited in the GenBank database under the accession number DQ025752 [GenBank: DQ025752].

## Authors' contributions

All authors contributed substantially to the study, and all have read and approved the final manuscript.
